# Presidential Oration: The 18^th^ Annual Conference of the Indian Academy of Neurology, Trichi, Tamil Nadu, September 24-26, 2010, Epilepsy Care in Developing Countries

**DOI:** 10.4103/0972-2327.74184

**Published:** 2010

**Authors:** Kurupath Radhakrishnan

**Affiliations:** R. Madhavan Nayar Center for Comprehensive Epilepsy Care, Sree Chitra Tirunal Institute for Medical sciences and Technology, Trivandrum, Kerala, India

**Keywords:** Developing countries, epilepsy, epilepsy surgery, treatment gap

## Abstract

Nearly 80% of the 50 million people with epilepsy worldwide reside in developing countries that are least equipped to tackle the enormous medical, social and economic challenges posed by epilepsy. These include widespread poverty, illiteracy, inefficient and unevenly distributed health care systems, and social stigma and misconceptions associated with epilepsy. Several studies have reported that a large proportion of patients with epilepsy in developing countries never receive appropriate treatment for their condition, and many, though diagnosed and initiated on treatment, soon discontinue treatment. Unaffordable cost of treatment, unavailability of antiepileptic drugs, and superstitious and cultural beliefs contribute to high epilepsy treatment gap in resource-poor countries. A significant proportion of the current burden of epilepsy in developing countries can be minimized by educating the public about the positive aspects of life with epilepsy and the primary and secondary physicians about current trends in the management of epilepsies, scaling up routine availability of low-cost antiepileptic drugs, and developing cost-effective epilepsy surgery programs.

## Introduction

It is indeed a great honour to serve the Indian Academy of Neurology as the President for the last one year. For the last several weeks, I have been pondering on as to what message I want to get across to the esteemed members of the Academy, junior and senior, at the end of my tenure as its President. Some of you might have read the inspiring novel “*The Last Lecture*” by Randy Pausch, a real life story of a computer science professor. Randy opines that the last lecture should convey, based on the analysis of one’s own life experiences, the following:

“*If we were to vanish tomorrow, What would we want to be remembered as our legacy? and What wisdom would we want to impart to our junior colleagues?*”

I started my career in neurology at the Postgraduate Institute of Medical Education and Research (PGIMER), Chandigarh, one of the foremost institutes of national importance for postgraduate studies in our country. I was fortunate enough to be mentored by Professor Jagjit Singh Chopra, a great teacher and researcher, the then Head of the Department of Neurology, PGIMER, Chandigarh. What can be more satisfying for me than the fact that I am delivering today this oration as the President of the organization founded by my own mentor? It is from Professor Chopra that I learned how one can effectively amalgamate the three functions as doctors we have to undertake, namely, patient care, teaching and research. During my stay in Medical University, Benghazi, Libya, I became interested in neuroepidemiology, the study of the distribution and determinants of neurological diseases in the community. The outstanding publications emanated from the studies conducted there came to the notice of the late Leonard Kurland of the Mayo Clinic, Rochester, MN, USA, who is often refereed to as the “*Father of Neuroepidemiology*”. Len taught me the art of how to meticulously select, plan and execute clinical research, and unambiguously communicate the results to the medical community. I was fortunate to receive training in electroencephaloghraphy from Donald Klass of the Mayo Clinic, the most reputed living legend in electroencephaloghraphy. The training in electroencephaloghraphy and epilepsy from the Mayo Clinic shaped my subsequent career. When I returned to India in 1994 and joined as the Head of the Department of Neurology, Sree Chitra Tirunal Institute of Medical Sciences and Technology, Trivandrum, these multinational experiences helped me to shape my career as a neurologist, neuroepidemiologist and epileptologist, and in establishing the R. Madhavan Nayar Center for Comprehensive Epilepsy Care. I hope through this paragraph, dealing briefly with my autobiography, I could convey the driving principle that has helped me to achieve what I am today. To quote from Mathatma Gandhiji, the “*Father of our Nation*”:

“*Men often become what they believe themselves to be. If I believe I cannot do something, it makes me incapable of doing it. But when I believe I can, then I acquire the ability to do it even if I didn’t have it in the beginning*”.

I wish to thank my colleagues in the R. Madhavan Nayar Center for Comprehensive Epilepsy Care for the help they rendered throughout the last 15 years. Our success is due to team approach, which means “*more of we and less of me*”.

## The Burden of Epilepsy

Epilepsy is a major public health problem as it affects an estimated 50 million people worldwide, and an additional 500 million people are involved as family members and care givers of the afflicted. Nearly 80% of the burden of epilepsy worldwide is borne by the resource-poor developing countries.[1] Developing countries (low-income and middle low-income countries according to World Bank listing) share several traits that make them least equipped to tackle the enormous medical, social and economic challenges posed by epilepsy. The health care systems are typically weak and inefficient. Poverty is widespread. The infrastructure and financial, human and material resources in the health sector are unevenly distributed. The few efficient health care facilities in these countries often benefit only the people residing in urban areas and those belonging to the economically advantaged section, and do not benefit the poor segment of the population mostly living in rural areas. Social stigma, myths and misconceptions associated with this disease often prevent people with epilepsy from seeking medical help. In most of these countries, patients and their care givers have to bear the entire cost of treatment.

In developed countries, the lifetime prevalence rates of epilepsy per 1000 person-years range from 3.5 to 10.7, and the incidence rates per 100,000 person-years range from 24 to 53.[2,3] In recent systematic reviews, the lifetime prevalence of active epilepsy per 1000 varied from 1.5 to 14 in Asia,[4] from 5.1 to 57.0 in Latin America,[5] and from 5.2 to 74.4 in sub-Saharan Africa.[6] These wide variations within and between these geographical regions may be apparent (attributable to misdiagnosis, varying definitions of epilepsy, failure to take account of the disease activity, and inconsistent definition of active epilepsy) or real (related to geographically relevant risk factors such as poverty, illiteracy, poor sanitation, inaccessibility of medical care, birth- and accident-related head trauma, and cerebral cysticercosis).[1] Through a three-phased survey (screening, diagnostic and confirmation phases), conducted in a semiurban area of central Kerala, southern India, the author obtained an age-adjusted prevalence rate of 4.7 per 1000.[7] Based on a prevalence rate of 5 per 1000 and an incidence rate of 50 per 100,000 per year, India with over 1 billion inhabitants will have anytime at least 5 million people with active epilepsy, to which nearly 500,000 get added annually. An ILAE/IBE/WHO study estimated the prevalence of epilepsy in rural China to be 4.6 per 1000.[8] China with over 1.3 billion people will have nearly 6 million people with epilepsy. China and India, the two most populous nations, together contribute to ~20% of people with epilepsy worldwide.

The diagnosis of epilepsy is fundamentally a clinical judgement based on history. The accuracy of the diagnosis of epilepsy depends on the skill and experience of the physician and the quality and reliability of the information provided by the witness. The ratio of neurologists to population varies in resource-poor countries from one neurologist to 3–5 million people, and in many Latin American and African regions, there are no neurologists at all.[9] India, with ~1000 neurologists, has one neurologist to 1 million people, which translates into one neurologist to care for 5000 persons with epilepsy. In a recent ILAE/IBE/WHO survey, although epilepsy specialists were available to provide care for people with epilepsy in 89% of high-income countries, they exist in only 56% of low-income countries.[10] Furthermore, whereas nearly two-thirds of people in developing countries reside in rural areas, nearly all the neurologists practice at or close to big cities and towns.[11] Therefore, a majority of persons with epilepsy in these countries are diagnosed, treated and followed up by primary and secondary care physicians without specific training or expertise in epilepsy management.

## Medical Treatment Gap

Several studies have reported that a large proportion of patients with epilepsy in developing countries do not receive appropriate treatment for their condition, a phenomenon known as the treatment gap.[12,13] The treatment gap is defined as the number of people with active epilepsy not on treatment or on inadequate treatment, expressed as a percentage of the total number with active epilepsy.[12] A recent systematic analysis which investigated the magnitude of treatment gap in resource-poor countries gave an overall rate of 56% (95% CI: 31–100%),[13] and the region and location specific rates were as follows: Latin America 55% (39–79%), Asia 64% (24–100%), Africa 49% (14–100%), rural 73% (50–100%), and urban 47% (34–64%). The authors ascribed the wide variations in the estimates to nonuniform definition of treatment gap and methods of its estimation. In the highly literate population of Kerala, southern India, the present author observed a treatment gap rate of 38% (35–41%).[7] In the recent systematic review, the causes of treatment gap expressed as median and range were cost of treatment 62% (11–90%), nonavailability of antiepileptic drug (AED) 53% (18–44%), belief in traditional treatment 44% (6–82%), and superstitions and cultural beliefs 40% (7–65%).[13]

In developing countries, a large proportion of patients with epilepsy, though diagnosed and initiated on AED treatment, soon discontinue the treatment. In epidemiological surveys, such patients would be categorized as not being on treatment. Das *et al*,[14] has coined the term “secondary treatment gap” to designate this phenomenon. In their prospective observation on 1450 patients followed up in an urban clinic in northeastern India, 620 (43%) discontinued their treatment within 1 year.[14] Among them, 89% had more than two breakthrough seizures following AED discontinuation. Inability to afford the treatment and lack of information about the consequences of medication noncompilance were the principal reasons for AED discontinuation. In an economic analysis set out to establish the cost-effectiveness of first-line AEDs (phenobarbitone, phenytoin, carbamazepine and valproic acid), it was concluded that a significant proportion of the current high treatment gap in resource-poor countries can be considerably minimized by scaling up the routine availability of low-cost AEDs like phenobarbitone and phenytoin.[15] Unfortunately, majority of patients with epilepsy in resource-poor countries are treated with multiple and often expensive AEDs simultaneously. In a study undertaken by the author, 58% of 972 patients were receiving polytherapy with AEDs at the time of referral to a tertiary care center from primary and secondary care facilities.[16] Among them, 95% received inadequate dosage of AEDs. There are two major reasons for this irrational polytherapy: widespread availability of AEDs (including new AEDs) in recent years and inadequate knowledge of primary and secondary care physicians (who initially treat majority of patients with epilepsy in developing countries) about the current trends in the pharmacotherapy of epilepsy. One of the most important jobs of the epilepsy specialists at the tertiary referral centers in developing countries should be the continuing medical education of the medical practitioners down the line.

## Surgical Treatment Gap

Nearly one-third of patients with newly diagnosed epilepsies on long-term follow-up will have their seizures unsatisfactorily controlled by treatment with AEDs.[17] The remarkable advances in neuroimaging technologies during the past two decades have allowed detection of a variety of brain lesions in over half of the patients with drug-resistant focal epilepsies, such as hippocampal sclerosis, malformations of cortical development, benign neoplasms, vascular malformations and focal gliotic lesions, that are amenable to surgical resections.[18] The understanding that a majority of patients with substrate-directed intracranial lesions associated with chronic focal epilepsies can be selected for surgery based on relatively simple and affordable non-invasive presurgical evaluation strategies has resulted in the creation of epilepsy surgery programs in developing countries in the recent years.[18] However, in a recent ILAE/IBE/WHO survey, epilepsy surgery was found to be available in only 13% of low-income countries compared to 66% of high-income countries.[10]

Epilepsy surgery centers in resource-poor countries lack the full range of state-of-the-art technologies usually available in the developed world to perform presurgical evaluation and surgery. Although the total direct cost of presurgical evaluation and surgery in resource-poor countries amounts to a small fraction of the cost incurred in the developed countries, this expenditure is beyond the reach of the majority.[19] Very few patients in resource-poor countries can afford the cost of intracranial electrodes used in invasive evaluation. In order to become cost-effective, epilepsy surgery centers in developing countries will have to achieve excellent results by selecting candidates destined to have seizure-free outcome with the locally available expertise and relatively inexpensive and non-invasive technologies, and without compromising on patient safety.[19] Patients with mesial temporal lobe epilepsy and those with circumscribed potentially epileptogenic lesions belong to this category. Patients with large epileptogenic lesions involving primarily one hemisphere, and those with diffuse encephalopathies and multifocal diseases can be selected for hemispherectomy and corpus callosotomy, respectively.[20,21]

Despite these challenges, in the last one and a half decades, several epilepsy surgery centers in developing countries have successfully implemented epilepsy surgery programs and produced results comparable to those from developed countries.[19,22] The R. Madhavan Nayar Center for Comprehensive Epilepsy Care at Trivandrum, Kerala, one of the leading centers for epilepsy care in Asia, has undertaken over 1200 epilepsy surgeries during the period of last 15 years. However, in India with over 500,000 potential epilepsy surgery candidates, not more than 200 epilepsy surgeries per year are being undertaken today.[23] This gap can be minimized only by developing many more cost-effective epilepsy surgery programs in different parts of the country.

## Social Issues

Throughout the world, misunderstanding and the resulting social stigma often cause more suffering to a person with epilepsy than the seizures themselves. The progressive emergence of positive public attitudes toward persons with epilepsy has been demonstrated in recently conducted knowledge, attitude practice surveys in developed countries.[24–26] Unfortunately, however, in the developing countries, epilepsy continues to be a highly stigmatizing condition.[4,7,27,28] For example, the percentage of respondents who thought epilepsy was a form of mental illness, who objected to their children paying with a child with epilepsy, and who objected to employing a person with epilepsy among the highly literate population of Kerala, southern India, were 27, 11, and 44%, respectively,[7] as compared to 3, 6, and 9% that existed in the United States 30 years ago.[24]

The psychosocial consequences of the stigma potentials of epilepsy in developing countries are nowhere more evident than in the case of women with epilepsy of the marriageable age. Unlike western culture, in most Asian countries, it is the responsibility of the parents to find a suitable match for their daughter and arrange marriage. Parents of a woman with epilepsy often get her married without informing the spouse and family of the disease.[29–31] Seizure exacerbation often occurs soon after marriage because of non-compliance to AED. Divorce ensues when the presence of epilepsy becomes evident.[29–31] Among 82 women with the onset of epilepsy prior to their marriage studied by the author, 55% concealed and 45% disclosed the history of epilepsy during marriage negotiations.[31] The frequency of divorce, separation and disturbed married life were significantly more in those who concealed. Significantly more patients who disclosed were employed compared to those who concealed.[31] Being employed is important for a woman with epilepsy as it makes her less dependent on the spouse and family on financial matters, and more confident in making independent decisions.

## Pragmatic Solutions

In 1997, the WHO, in collaboration with ILAE and IBE, launched the Global Campaign Against Epilepsy to improve the care of people with epilepsy in resource-poor countries.[32] To test the campaign’s main goal of reducing the treatment gap for epilepsy, demonstration projects were set up in a few locations in resource-poor countries.[33] The results from the largest of these projects from rural China, which enrolled 2455 patients, revealed that primary care physicians with basic training could very effectively treat people with epilepsy with phenobarbital administered once daily at night.[34] The diagnosis of convulsive epilepsy, confirmed by a local neurologist, was based on the history and on a witness account without the aid of EEG. Nearly three-fourths of patients who completed 24 months’ treatment achieved at least 50% reduction seizure frequency and a quarter of patients were seizure-free. Only few cognitive or behavioral adverse effects were observed. The probability of retention on phenobarbital treatment was 84% at 1 year and 76% at 2 years.[34] The results of the Yelandur study conducted by the late Dr. K. S. Mani, the “*Father of Indian Epileptology*” were nearly identical.[35]

A model for epilepsy care in resource-poor countries from community to national levels should take into consideration the heterogeneity of epileptic disorders and a need-based approach to their management. While a majority of the patients with epilepsy can be treated at the primary or secondary care facilities, few with difficult to control epilepsies will require referral to tertiary and comprehensive epilepsy care facilities. To improve care at all levels, a close interaction between general practitioners, physicians, neurologists and epileptologists, a partnership between governmental and non-governmental health care agencies, and help from high-income countries in training epilepsy specialists in advanced diagnostic and therapeutic techniques are essential. Several WHO/ILAE/IBE recommended epilepsy care initiatives are being successfully implemented in the recent years by national and regional chapters and governmental and non-governmental organizations. For example, the Indian Epilepsy Society recently brought out Guidelines for the Management of Epilepsy in India (GEMIND) focusing on practical issues that will aid general practitioners in diagnosing epileptic seizures, initiating the most appropriate AED and in making need-based decision to refer a patient for specialized care.[36]

## Conclusions

Nearly 80% of the global burden of epilepsy is borne by the resource-poor developing countries, which are least equipped to tackle the enormous medical, social and economic challenges posed by this condition. Consequently, a large proportion of patients with epilepsy in resource-poor countries never receive appropriate treatment for their condition, and many, though diagnosed and initiated on treatment, soon discontinue the treatment. The current high treatment gap in developing countries can be considerably minimized by scaling up the routine availability of low-cost AEDs like phenobarbitone. Because of the marked scarcity of comprehensive epilepsy care centers, only a miniscule of patients with difficult to control epilepsies in resource-poor countries ever get a chance to undergo presurgical evaluation and surgery. If the care of persons with epilepsy in developing countries has to improve at the community level, undergraduate medical curriculum should have more emphasis on this common disorder, primary and secondary care physicians need to be regularly educated about the recent advances in its management, low-cost AEDs should be made available to poor patients free of cost, and cost-effective epilepsy surgery programs should be developed in selected centers. A comprehensive epilepsy care model should take into consideration the marked heterogeneity of the disorder and its variable impact on the patient, family and community.
